# CD44 Is Associated with Poor Prognosis of ccRCC and Facilitates ccRCC Cell Migration and Invasion through HAS1/MMP9

**DOI:** 10.3390/biomedicines11072077

**Published:** 2023-07-24

**Authors:** Tan Du, Zonglong Wu, Yaqian Wu, Yunchong Liu, Yimeng Song, Lulin Ma

**Affiliations:** Department of Urology, Peking University Third Hospital, Beijing 100191, China; jeckiedo@foxmail.com (T.D.);

**Keywords:** ccRCC, CD44, HAS1, MMP9, cancer metastasis

## Abstract

Background: In many solid tumors, CD44 has been identified as a cancer stem cell marker as well as an important molecular in cancer progression and metastasis, making it attractive for potential therapeutic applications. However, our knowledge of the biological function and mechanism of CD44 in clear cell renal cell carcinoma (ccRCC) is limited. Methods: In this study, the expression, prognostic values and functional enrichment analysis of CD44 in ccRCC were analyzed using public databases. Quantitative real-time PCR (qRT-PCR), Western blotting, and immunohistochemical (IHC) assays were taken to detect CD44 expression in ccRCC tissues. The effects of CD44 on the proliferation, migration and invasion of ccRCC cells were investigated by gain-of-function and loss-of-function experiments. Subcutaneous models further confirmed the role of CD44 in tumor growth. The relationship between CD44, HAS1 and MMP9 was investigated to uncover the regulatory mechanism of CD44 in ccRCC. Results: CD44 was significantly upregulated in ccRCC and associated with poor overall survival (OS). Based on the functional enrichment analysis and PPI network, we found that CD44 had associations with ECM interaction and focal adhesion pathway. Clinical ccRCC sample validation revealed that CD44 mRNA and protein expression were significantly increased in ccRCC tissues, and strong CD44 staining was observed in four metastatic ccRCC cases. In vitro experiments showed that CD44 overexpression promoted cell proliferation, migration and invasion. In vivo experiments also demonstrated that CD44 overexpression accelerated tumor formation in mice. Finally, we found that CD44 regulates the expression of HAS1 in ccRCC, which is essential for the secretion of MMP9 and cell migratory ability. Conclusion: The upregulation of CD44 mRNA and protein expressions in ccRCC is indicative of unfavorable clinical prognoses. The CD44/HAS1/MMP9 axis is believed to exert a significant influence on the regulation of ECM degradation and ccRCC metastasis.

## 1. Introduction

Renal cell carcinoma (RCC) is a primary malignant tumor that originates from the renal epithelium and accounts for 90% of kidney cancer [1]. Clear cell renal cell carcinoma (ccRCC) represents the most prevalent pathological subtype of RCC, characterized by a high propensity for metastasis and immune infiltration, and is associated with a poorer prognosis compared to other RCC subtypes [2]. The limited efficacy of conventional therapies and acquired resistance to targeted treatments have contributed to the low overall survival rate of ccRCC patients, making it the primary cause of kidney cancer-related mortalities [3]. At the time of initial diagnosis, around 20% to 30% of patients with ccRCC exhibit a concomitant presence of distant metastases, while over one-third of ccRCC patients develop metastases following surgical resection [4,5]. Hence, comprehending the fundamental mechanisms of ccRCC cell metastasis can facilitate the development of more efficacious therapeutic approaches for ccRCC.

Cluster of differentiation 44 (CD44) is a cell surface transmembrane glycoprotein that is expressed in various human cell types and has been extensively studied in relation to its involvement in physiological processes, as well as its significant roles in tumorigenesis and cancer metastasis [6,7]. In particular, elevated CD44 expression has been observed in breast, colon, prostate, ovarian, and gastric cancer and is associated with aggressive biological behaviors and a diminished prognosis [8,9,10,11,12]. Tumor cells overexpressing CD44 demonstrate numerous characteristics of cancer stem cells (CSCs), including the ability to self-renew, high potential for tumor initiation, capacity for epithelial–mesenchymal transition (EMT), and resistance to chemotherapy [13,14]. CD44 interacts with various extracellular matrix (ECM) constituents within the tumor microenvironment, with hyaluronic acid (HA) being the primary ligand for CD44 [15]. The binding of HA and CD44 triggers the activation of multiple signaling pathways that regulate cell adhesion, proliferation, and migration, thereby modulating cytoskeletal dynamics and promoting cell survival [16]. Previous immunohistochemical studies have demonstrated that CD44 expression in RCC was notably elevated, and augmented expression of CD44 and MMP-9 was positively associated with metastatic ccRCC [17,18]. 

ccRCC tumors have the potential to develop into tumor thrombus (ccRCC-TT) within the renal vein or inferior vena cava, which is indicative of a poor prognosis and a more aggressive population of ccRCC cells [19]. In a previous study, we identified a set of differentially expressed genes between primary ccRCC tumors and thrombus. Our findings revealed that 25 genes were significantly upregulated in ccRCC-TT, including *HAS1* and *MMP9* [19]. HAS1 is the main enzyme responsible for hyaluronan (HA) production, which is the most specific ligand for CD44 activation [20]. MMP9 has the ability to degrade the ECM components and plays a crucial role in cancer invasion, and it is frequently co-expressed with CD44 in ccRCC [17,21]. A prior investigation demonstrated that CD44 partially regulates MMP2 expression in an HA-dependent manner [22]. Whether CD44 can mediate cell migration and MMP9 expression in ccRCC cells by improving HAS1 is a new direction proposed by this study.

Currently, our study explores the function of CD44 in ccRCC and elucidates its underlying mechanism. Through bioinformatic analysis and validation of ccRCC samples, we have observed an elevation in CD44 expression at both protein and transcript levels in ccRCC. Furthermore, our findings indicate that high CD44 expression is associated with reduced overall survival in ccRCC patients. Additionally, we have demonstrated that CD44-induced upregulation of HAS1 promotes cell migration, invasion, and MMP9 expression, thereby contributing to ccRCC tumorigenesis and progression. These results underscore the role of CD44 in promoting tumor metastasis in ccRCC and highlight its active regulation of HAS1 expression.

## 2. Material and Methods

### 2.1. Bioinformatics Analysis

RNA-sequencing (RNA-seq) data of 523 clear cell renal cell cancer tissues and 100 adjacent normal tissues, including the clinical information of the kidney renal clear cell carcinoma (KIRC) cohort, were downloaded from the TCGA database. The protein expression of CD44 in CPTAC database was assessed by ULCAN (http://ualcan.path.uab.edu/analysis.html (accessed on 14 May 2022). The PPI network was constructed using STRING (https://string-db.org/ (accessed on 14 May 2022). CD44 was entered in the STRING online tool, and an interaction score greater than 0.4 of related genes was selected for analysis. Subsequently, Gene Ontology (GO) and Kyoto Encyclopedia of Genes and Genomes (KEGG) pathway enrichment analysis of CD44-related genes were constructed using cluster Profiler Package in the R.

### 2.2. Clinical ccRCC Samples

ccRCC tissues and corresponding adjacent normal tissues were collected from the Department of Urology, Peking University Third Hospital. Our study was approved by the Institutional Review Board of Peking University Third Hospital, and written informed consent was obtained from all patients prior to our study. All patients underwent standard resection. The human ccRCC tissue microarray (TMA) chips were procured from Shanghai Outdo Biotech Co., Ltd. (Shanghai, China). The human ccRCC tissue cDNA array was procured from Shanghai Outdo Biotech Co., Ltd. (Shanghai, China).

### 2.3. Immunohistochemistry

The TMA was subjected to incubation at 60 °C for 2 h. Subsequently, the chips were dewaxed thrice in xylene and dehydrated in a series of graded alcohols. The chips were then microwaved in a pH 6.0 citric acid buffer to facilitate antigen retrieval. To mitigate endogenous peroxidase activity, the chips were treated with 0.3% H_2_O_2_ for 15 min at room temperature. After preincubation with 10% normal goat serum to block nonspecific sites for 30 min, the chips were incubated with primary antibodies at 4 °C overnight, followed by HRP-conjugated secondary antibodies. Subsequently, the sections underwent three 5-minute rinses with PBS, followed by the development of reactions utilizing diaminobenzidine tetrahydrochloride (DAB) as a substrate. Hematoxylin (Invitrogen, Carlsbad, CA, USA) was employed for counterstaining cellular nuclei, and neutral gum was utilized for sealing the sections. The IHC primary antibody used in this study was anti-CD44 (CST, Danvers, MA, USA, Mouse mAb #3570).

### 2.4. ccRCC Cell Lines

Human ccRCC cell lines 786-O and A498 were purchased from the American Type Culture Collection (ATCC). Cells were cultured in RIPA-1640 medium (Gibco, CA, USA, 16600082). All media contained 10% FBS (Gibco,10270106) and 1% Penicillin–Streptomycin solution (Sigma, St. Louis, MO, USA, V900929). All cell lines were incubated at 37 °C in a humidified atmosphere with 5% CO_2_.

### 2.5. Lentivirus and Cell Transfection

The synthesis of CD44 overexpression lentivirus (CD44 OE), CD44 shRNA lentivirus, and corresponding control lentiviruses (shNC) was carried out by Hanbio Tech (Shanghai, China). Subsequently, the lentivirus was transfected into 786-O and A498 cells using polybrene (MCE, Monmouth Junction, NJ, USA, HY-112735). Following transfection, the cells were cultured for 48 h and subsequently selected with 2 μg/mL puromycin (Solarbio, Beijing, China, P8230). The siRNA targeting HAS1 was designed and synthesized by SyngenTech, Beijing, China. siRNA transfection was conducted by Lipo3000 transfection reagent (Thermo Fisher, Waltham, MA, USA, L3000015).

### 2.6. Colony Formation Assay

A total of 200 cells were seeded per well in a 6-well plate and cultured in complete medium at 37 °C with 5% CO_2_ for a duration of two weeks. Following this, the colonies were immobilized using 4% paraformaldehyde and subsequently stained with 0.1% crystal violet for a period of 30 min. The surplus staining solution was meticulously eliminated using distilled water. Results were presented as mean ± SD of three independent experiments.

### 2.7. Cell Counting Kit-8 (CCK-8) Assay

Cell viability was assessed through the utilization of a Cell Counting Kit-8 (CCK-8) assay (Beyotime, Shanghai, China, C0037) in accordance with the manufacturer’s guidelines. Specifically, cells were seeded at a density of 5 × 10^3^ cells/well in a 96-well plate. Subsequently, a medium containing 10% Cell Counting Kit-8 was introduced to each well at various time intervals (0, 24, 48, and 72 h) and incubated at 37 °C for 1 h. The optical density was measured at 450 nm using a spectrophotometer.

### 2.8. Transwell Migration and Invasion Analysis

Cell migration and invasion capabilities were assessed using a Transwell plate (Corning, NY, USA, 3422). Following a 24 h period of starvation, cells (1 × 10^5^) suspended in serum-free medium were introduced into the upper chamber of the Transwell plate, which was either precoated with Matrigel (BD Bioscience, San Jose, CA, USA) or left uncoated. The lower chambers were filled with 600 μL of complete medium containing 10% serum. After being cultured for 12–24 h, cells that had traversed the membranes were fixed with 4% paraformaldehyde and stained with 0.1% crystal violet. The migrated and invasive cells were subsequently observed under a microscope. Results were presented as mean ± SD of three independent experiments.

### 2.9. Real-Time Quantitative RT-PCR

The human ccRCC tissue cDNA array was procured from Shanghai Outdo Biotech Co., Ltd. (Shanghai, China). Total RNA was isolated from human ccRCC cells using TRIzol reagent (Invitrogen, Waltham, MA, USA), and 1 μg total RNA was reversely transcribed into the first-strand cDNA with a reverse transcription kit (Yeasen, Shanghai, China). Subsequently, the acquired cDNA was used as a template for quantitative real-time PCR analysis using the SYBR Green Premix (Yeasen, Shanghai, China) on a 7500 sequence detection system (Applied Biosystems, Carlsbad, CA, USA). The relative RNA expression levels were calculated using the 2^−ΔΔCT^ method with GAPDH as an endogenous control. The primers used in this study were: CD44-F: CTGCCGCTTTGCAGGTGTA; CD44-R: CATTGTGGGCAAGGTGCTATT; HAS1-F: TCAAGGCGCTCGGAGATTC; HAS1-R: CTACCCAGTATCGCAGGCT.

### 2.10. Western Blotting Analysis

The cells were collected, rinsed, and dislodged in cold RIPA lysis buffer. Following a 20-minute lysis period, proteins were extracted and their concentrations were determined using a BCA assay kit (Thermo Fisher, Waltham, MA, USA, 23227). The samples were then subjected to sodium dodecyl sulfate-polyacrylamide gel electrophoresis, transferred onto PVDF membranes (Millipore, Billerica, MA, USA), and blocked with 8% fat-free milk for one hour at room temperature. After washing with TBST, the membranes were incubated with primary antibodies at 4 °C overnight. On the following day, the membranes underwent three washes in TBST prior to the introduction of HRP-labeled secondary antibodies. The signals were subsequently identified through the utilization of a chemiluminescent HRP substrate (Millipore) on the Bio-Rad imaging system (Bio-Rad, Hercules, CA, USA). The antibodies utilized in this study were: anti-CD44 (CST, Danvers, MA, USA, Mouse mAb #3570), anti-HAS1(abcam, Cambridge, MA, USA, ab198846), anti-MMP9(abcam, Cambridge, MA, USA, ab38898), and anti-GAPDH (proteintech, Wuhan, China, 60004-1-Ig).

### 2.11. Animal Experiments

Balb/C nude mice, aged 6–7 weeks, were procured from Charles River Laboratories (Beijing, China) and housed in a specific pathogen-free environment. For subcutaneous xenograft experiments, approximately 2 × 10^6^ ccRCC cells were subcutaneously injected into the left flank of the mice (*n* = 4 per group). Tumor sizes were measured every 7 days, which were calculated using the formula: length × width^2^ × 0.5. Following a period of 7 weeks, the mice were euthanized, and the tumors were surgically removed. 

### 2.12. Statistical Analysis

The present study utilized three independent replicate experiments to generate all data. Results were presented as means ± SEM and subjected to statistical analysis using GraphPad Prism 8.0. Student’s *t*-test was employed to compare two groups, with statistical significance set at *p* < 0.05. Correlation analysis was analyzed by Pearson correlation. The level of significance was denoted by asterisks as follows: *, *p* < 0.05; **, *p* < 0.01; ***, *p* < 0.001; ****, *p* < 0.0001.

## 3. Results

### 3.1. Elevated Expression of CD44 Is Indicative of an Unfavorable Prognosis among Patients with ccRCC

**Figure 1:**
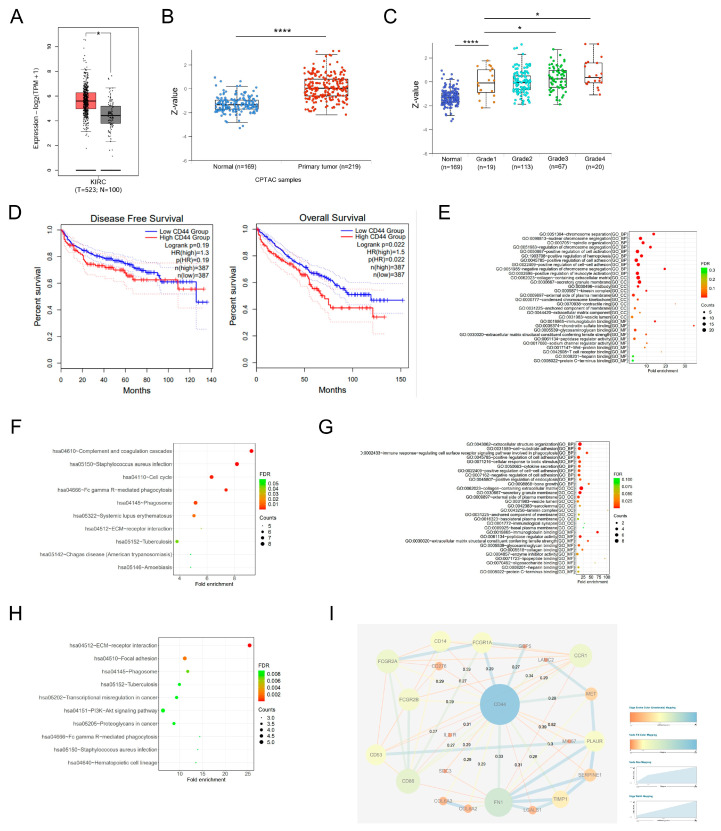
Bioinformatic analysis of the expression and prognostic value of CD44 in ccRCC. (**A**) Analysis of CD44 mRNA expression in TCGA-KIRC. The red box represents tumor tissues and the grey box represents normal tissues. (**B**) Analysis of CD44 protein expression in CPTAC-ccRCC. The red dots represent ccRCC tissues and the grey dots represent normal tissues. (**C**) The correlation between CD44 expression and ccRCC tumor stage in CPTAC-ccRCC. (**D**) Relationship between CD44 expression and disease-free survival (left) and overall survival (right). (**E**,**F**) GO and KEGG functional enrichment analysis of 165 DEGs related to CD44. (**G**,**H**) GO and KEGG functional enrichment analysis of 22 genes screened from the PPI network that are most closely related to CD44. (**I**) The PPI network of CD44 and its 22 most frequently altered neighboring genes. * *p* < 0.05, **** *p* < 0.0001.

In order to conduct a comparison of CD44 expression between ccRCC tissues and normal kidney tissues, an initial assessment of CD44 transcriptional levels was performed on the TCGA-KIRC dataset. The results indicated a greater mRNA expression of CD44 in ccRCC tissues when compared to their corresponding normal tissues (Figure 1A). Concurrently, an elevated protein expression of CD44 was detected in ccRCC tumor tissues within the CPTAC cohort (Figure 1B), with a significant correlation between CD44 levels and the tumor stage of ccRCC patients (Figure 1C). To further conduct a more comprehensive assessment of the prognostic significance of CD44 in ccRCC, we performed an analysis of the relationship between CD44 expression and survival rates among ccRCC patients sourced from TCGA. Our findings indicate that patients with elevated CD44 expression levels exhibited poorer overall survival outcomes in comparison to those with lower CD44 expression levels. However, we did not observe any statistically significant correlation between CD44 expression and disease-free survival (Figure 1D). Through the integration of CD44 expression-related genes and differentially expressed genes (DEGs) in TCGA-KIRC, a total of 165 DEGs associated with CD44 were identified. Subsequent functional enrichment analysis revealed that these DEGs were involved in critical biological processes such as cell adhesion, cell cycle regulation, and ECM–receptor interaction (Figure 1E,F). In addition, we also performed pathway enrichment analysis on the 22 genes screened from the PPI network that are most closely related to CD44 (Figure 1I); the results showed that CD44 was significantly involved in the most significant pathways including ECM–receptor interaction and focal adhesion (Figure 1G,H). These results suggest that CD44 was up-regulated in ccRCC and had a positive correlation with unfavorable patient survival, potentially promoting ccRCC tumorigenesis by modulating cell-matrix interactions and ECM remodeling.

### 3.2. CD44 Is Overexpressed in ccRCC Samples and Is Significantly Correlated with Tumor Stage

**Figure 2:**
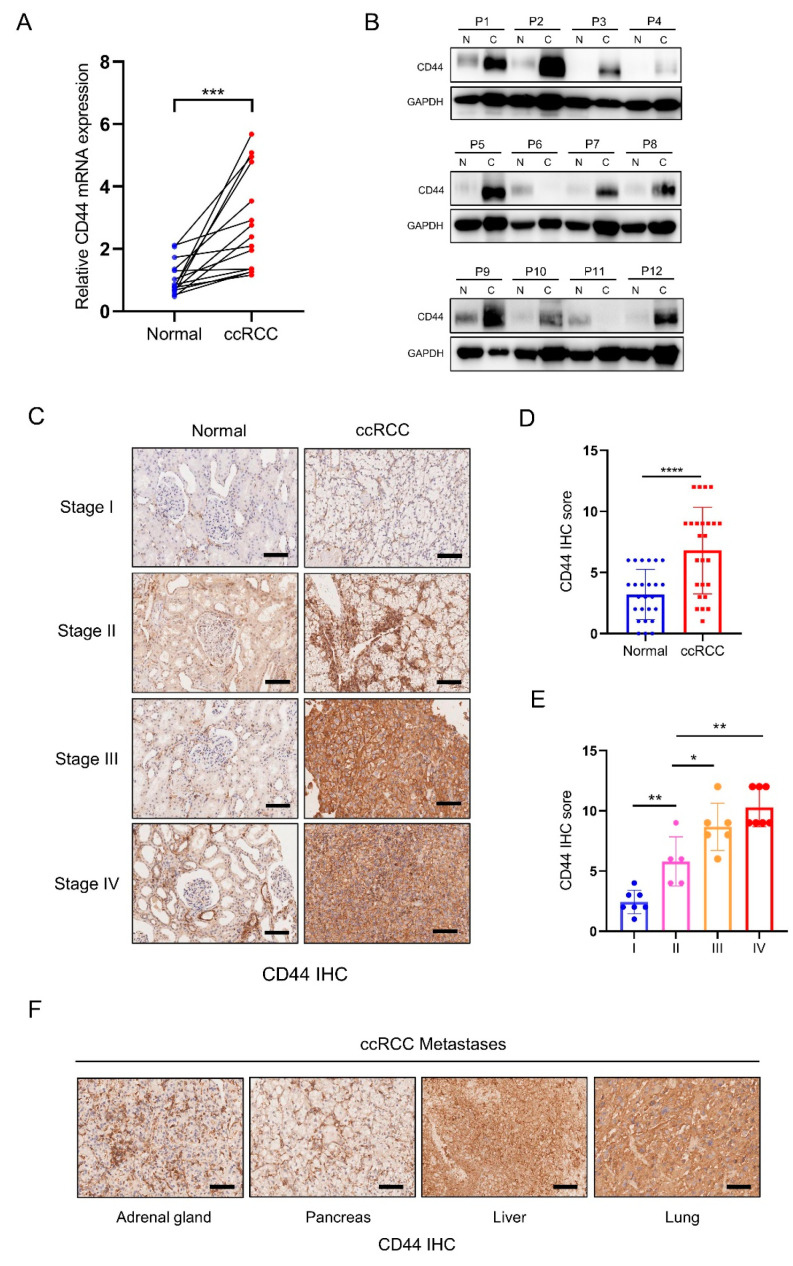
CD44 is up-regulated in ccRCC clinical samples. (**A**) The mRNA expression of CD44 in a human ccRCC cDNA array, including 15 paired normal and ccRCC tissues. (**B**) The protein expression of CD44 in 12 cases of ccRCC tumors (C) compared with their nontumor tissues (N). (**C**) Representative immunohistochemistry (IHC) images showing the staining intensity of CD44 in ccRCC from stage I to stage IV. Scale bars, 100 μm. (**D**) The statistical results of CD44 IHC in pared ccRCC tumor tissues are shown. (**E**) The correlation between CD44 IHC score and ccRCC tumor stage was shown. (**F**) Representative IHC images showing strong CD44 staining in four ccRCC metastasis tissues. Scale bars, 100 μm. * *p* < 0.05, ** *p* < 0.01, *** *p* < 0.001, **** *p* < 0.0001.

To validate CD44 expression in clinical samples of ccRCC, we conducted an analysis of CD44 mRNA expression in a human ccRCC cDNA array (*n* = 15) and assessed CD44 protein expression levels in paired ccRCC tissues and adjacent tissues (*n* = 12) obtained from advanced ccRCC patients. qRT-PCR results demonstrated a significant increase in CD44 mRNA expression in ccRCC tissues compared to noncancerous controls (Figure 2A). Additionally, Western blot analysis revealed a marked upregulation of CD44 protein expression in ccRCC specimens compared to adjacent healthy tissues (Figure 2B). Furthermore, immunohistochemistry analysis of ccRCC tissue microarray revealed the presence of CD44, which exhibited significantly higher expression levels in ccRCC compared to noncancerous tissues (Figure 2C,D). The intensity of CD44 staining was positively associated with the tumor stage (Figure 2C,E). Notably, strong CD44-positive staining was observed in four cases of ccRCC distant metastasis tissues, indicating a potentially critical role of CD44 in the metastatic colonization of ccRCC cells (Figure 2F). These findings from the validation of ccRCC samples further support our previous bioinformatics analysis.

### 3.3. CD44 Facilitates ccRCC Cell Proliferation, Migration, and Invasion In Vitro

**Figure 3:**
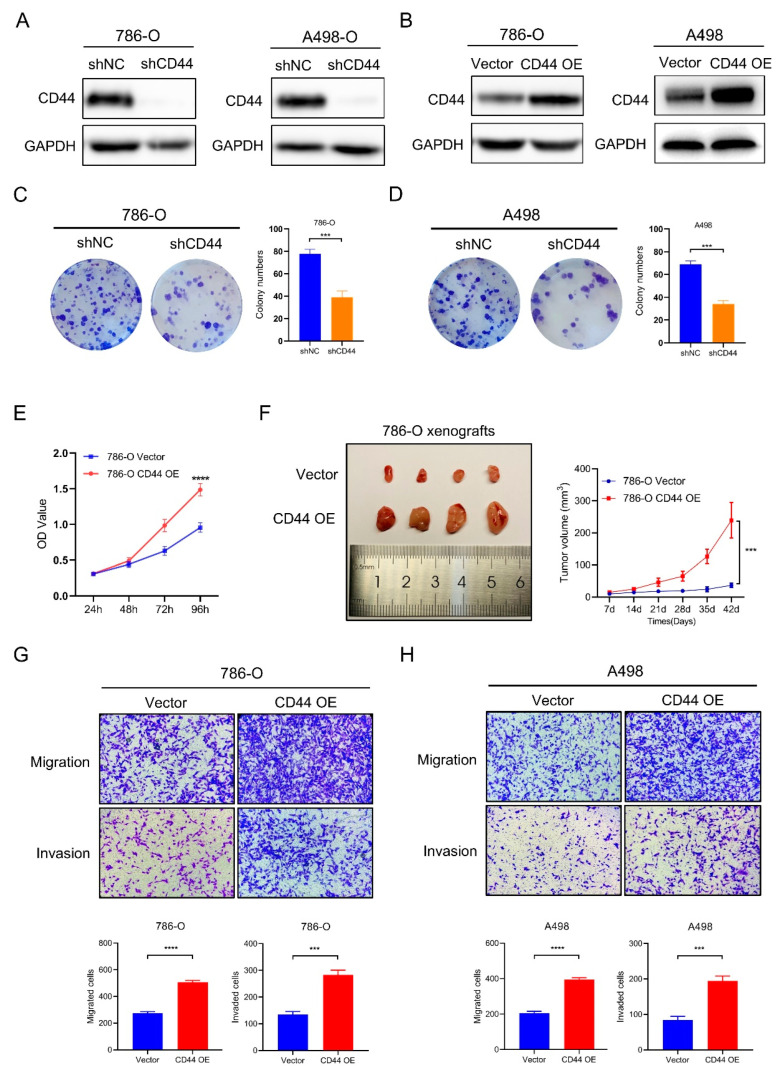
CD44 overexpression promotes the proliferation, migration and invasion of ccRCC cells. (**A**,**B**) The efficiency of CD44 knockdown (**A**) and overexpression (**B**) was confirmed using Western blot analysis. GAPDH as an internal control. (**C**,**D**) Cell proliferation was measured by using a colony formation assay. (**E**) CCK-8 assay was performed to detect cell proliferation of 786-O CD44 OE cells. (**F**) Representative images of tumor formation in Bulb/C mice injected with control (Vector) and CD44 overexpressing (CD44 OE) 786-O cells. Statistical results of tumor growth rate were presented at right. (**G**,**H**) Cell migration and invasion were analyzed using transwell migration and Matrigel invasion assay. Magnification: 100×. *** *p* < 0.001, **** *p* < 0.0001.

In order to examine the role of CD44 in tumorigenesis, we first investigated its impact on cell proliferation. Lentivirus transfection was utilized to establish stable CD44 knockdown (shCD44) and overexpression ccRCC (CD44 OE) cell lines. Western blot analysis confirmed the high efficiency of CD44 knockdown and overexpression in 786-O and A498 cells (Figure 3A,B). The results of the colony formation assay demonstrated that CD44 knockdown significantly inhibited ccRCC cell proliferation, as evidenced by a reduction in colony numbers in the 786-O and A498 shCD44 group (Figure 3C,D). Moreover, the CCK-8 assay validated a significant increase in cell proliferation ability subsequent to CD44 overexpression, as evidenced by the augmented growth rate of 786-O and A498 CD44 OE cells compared to control cells (Figure 3E, Supplementary Figure S1). Similar results were observed in the xenograft mice model, wherein the mice injected with 786-O control cells developed tumors, whereas CD44 overexpression substantially amplified the tumor proliferation rate (Figure 3F). The migratory and invasive capabilities of cancer cells are crucial determinants of cancer metastasis. In light of this, we conducted an investigation into the motility of ccRCC cells, utilizing transwell migration and invasion assays. Our findings indicate that 786-O and A498 CD44 OE cells exhibited significantly greater migratory potential in comparison to their respective control cells (Figure 3G,H). In the invasion assay, where the cells are allowed to transverse through Matrigel-coated membranes, the result revealed that the overexpression of CD44 led to a notable increase in the invasive ability of 786-O and A499 cells (Figure 3G,H). Conversely, CD44 knockdown was observed to impede the migration and invasion of 786-O cells (Supplementary Figure S2). The results mentioned above provide evidence that the increased expression of CD44 is strongly correlated with the malignant characteristics of ccRCC cells.

### 3.4. CD44 Regulates MMP9 in ccRCC by Up-Regulating HAS1 Expression

**Figure 4:**
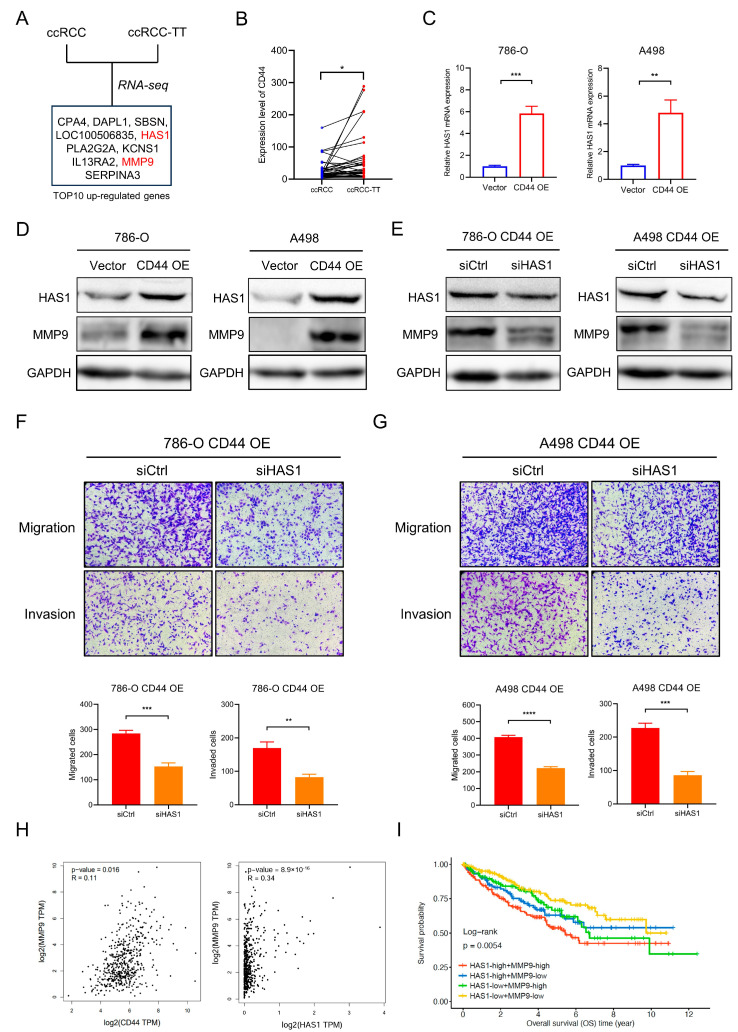
CD44 regulates MMP9 expression and cell invasion through HAS1. (**A**) The top 10 differentially up-regulated genes in ccRCC-TT in comparison to primary ccRCC tumors. (**B**). The mRNA expression of CD44 was upregulated in the ccRCC-TT. (**C**) qRT-PCR analysis showed HAS1 mRNA expression was increased in 786-O and A498 CD44 overexpression cells. (**D**) Western blot analysis showed the expression of HAS1 and MMP9 were up-regulated in CD44 overexpression cells. (**E**) Western blot analysis showed the expression of MMP9 was reversed by inhibiting HAS1 in CD44 overexpression cells (**F**,**G**). Transwell migration and invasion assay showed HAS1 inhibition suppressed ccRCC cell migratory abilities mediated by CD44 overexpression. (**H**) Correlation analysis of CD44/HAS1 and MMP9 mRNA expression in ccRCC patients from TCGA database. (**I**) Survival analysis of HAS1, MMP9 expression group in TCGA-KIRC. * *p* < 0.05, ** *p* < 0.01, *** *p* < 0.001, **** *p* < 0.0001.

The invasion of renal tumors into the venous system, resulting in the formation of tumor thrombus (TT), is a distinctive clinical feature of RCC that is observed in roughly 15% of RCC patients [23]. The degree of ccRCC-TT invasion drives tumor staging and leads to a worse prognosis. In our prior investigation, we effectively discerned 25 genes that exhibited higher expression levels in ccRCC-TT in comparison to its primary ccRCC tumors [19]. Remarkably, within this gene set, *HAS1* and *MMP9* emerged as two of the top ten significantly upregulated genes in ccRCC-TT (Figure 4A). Concurrently, the upregulation of CD44 expression was observed in ccRCC-TT (Figure 4B). HAS1 is responsible for Hyaluronan production, which is the most specific ligand for CD44 activation, and MMP9 is a matricellular protein associated with ECM remodeling; thus, we sought to investigate whether CD44 regulated MMP9 expression via HAS1, thereby resulting in tumor metastasis and invasion. qRT-PCR results indicate that the mRNA expression of HAS1 was elevated in CD44-overexpressing ccRCC cells (Figure 4C), conversely, the downregulation of CD44 resulted in a contrary trend in HAS1 expression (Supplementary Figure S3). Moreover, the overexpression of CD44 significantly augmented the protein expression of both HAS1 and MMP9 (Figure 4D). Interestingly, rescue experiments showed that the protein level of MMP9 was decreased after interfering with HAS1 (Figure 4E). Moreover, HAS1 inhibition profoundly reversed CD44-overexpression-mediated enhancement of cell metastatic capacity, evidenced by a decreased number of migrated and invading cells in the transwell assay (Figure 4F,G). These results suggest that HAS1 was regulated by CD44 and functionally involved in the increased expression of MMP9. The analysis of TCGA-KIRC demonstrated a positive correlation between the expression of CD44 and HAS1 with MMP9 (Figure 4H, Supplementary Figure S4). Subsequently, we categorized ccRCC patients based on the expression of HAS1 and MMP9, and survival analysis indicated that individuals exhibiting elevated levels of both HAS1 and MMP9 experienced the most unfavorable prognosis (Figure 4I).

## 4. Discussion

CD44 is a cell surface glycoprotein that has been extensively researched as a cancer-promoting molecule due to its crucial involvement in tumor development and progression [24]. Its expression is widespread in various carcinomas, and it regulates a range of cancer cell functions, including proliferation, adhesion, migration, angiogenesis, and cytoskeleton rearrangement [16]. Additionally, CD44 serves as a molecular marker for cancer stem cells (CSCs) and is frequently employed alone or in conjunction with other putative CSC markers to isolate CSCs from solid tumors through flow cytometry sorting [25,26]. The salient characteristics of CSCs encompass their capacity for self-renewal and tumor initiation, as well as their resistance to numerous chemotherapeutic agents, which contributes to drug resistance [27]. In addition to its regulatory functions in cell survival, aggregation, and cellular motility, recent research has revealed that CD44 plays a crucial role in the regulation of ferroptosis through the stabilization of SLC7A11 [28]. The multifaceted involvement of CD44 in various pathological activities within cancer cells necessitates comprehensive investigation into its biological function, thereby facilitating the development of novel therapeutic approaches for patients afflicted with high-CD44 tumors.

Renal cell carcinoma (RCC) is the predominant form of primary kidney cancer, comprising 2–3% of all cancer diagnoses and ranking third among urological-cancer-related fatalities globally [29]. RCC is further classified into multiple subtypes, with clear cell RCC (ccRCC) constituting 75% of RCC cases and exhibiting a greater propensity for aggressiveness and rapid metastasis compared to other RCC subtypes, such as papillary RCC (15%) and chromophobe RCC (5%) [30]. Previous immunohistochemical investigations have demonstrated that the presence of CD44 positivity in ccRCC may be linked to increased expression levels of MMP2 and MMP9, and a positive correlation was observed between the upregulation of CD44 and MMP-9 in metastatic ccRCC [17,18]. Another study revealed that heightened CD44 expression was associated with more aggressive tumor behavior, progression, and unfavorable overall survival outcomes in ccRCC patients, but not in those with papillary and chromophobe RCC [31]. Consistently, our study presents evidence that mRNA and protein expressions of CD44 were elevated in ccRCC tissues, in line with our analysis of TCGA-KIRC and CPTAC databases. Furthermore, intense CD44 IHC staining was observed in four metastasis tissues, and a positive correlation was found between CD44 expression, ccRCC tumor stage, and unfavorable patient survival.

The primary ligand of CD44 is hyaluronan, a significant constituent of the extracellular matrix (ECM). The co-localization of hyaluronan and CD44 has been observed in various cancers and has been demonstrated to facilitate tumorigenesis [32]. Hyaluronan, also known as hyaluronic acid (HA), is a large glycosaminoglycan synthesized by the HAS family, which includes HAS1, HAS2, and HAS3 [33]. The interaction between HA and CD44 is crucial for activating CD44, which triggers tumorigenic signaling pathways involved in tumor development, metastasis, and chemo-resistance [34]. Among three HAS isoforms, HAS1 is a transmembrane protein located in the cytoplasmic space to generate intracellular HA [33]. Several studies have demonstrated the significance of intracellular HA in cellular function, and it has been observed that CD44 can enhance HA synthesis by activating the transcription of the *HAS* gene [35]. In recent years, numerous studies have investigated the relationship between HAS1 and tumorigenesis, revealing that HAS1 plays a crucial role in malignant transformation and tumor growth [36,37]. In the TCGA cohorts, HAS1 is the only HAS isoform that could predict shorter survival of ccRCC patients, but the study of HAS1 in ccRCC is limited. Our prior research has identified *HAS1* and *MMP9* as members of the TOP 25 up-regulated genes in ccRCC thrombus in comparison to primary ccRCC tumors. This finding suggests that HAS1 could potentially exert a noteworthy influence on the aggressiveness of ccRCC and participate in its capacity to metastasize into renal vessels. By conducting in vitro cell experiments, we discovered that CD44 regulated HAS1 in ccRCC cell lines. The inhibition of HAS1 in CD44-overexpressing cells resulted in the reversal of MMP9 expression, and the inhibition of CD44 mediated cell migration and invasion. As a result, we postulate that CD44 may augment intracellular HA synthesis by upregulating HAS1 expression, which in turn could facilitate HA-CD44 binding and promote a pro-metastatic effect.

Matrix metalloproteinases (MMPs) are a family of zinc-dependent enzymes comprising over 25 distinct members. These enzymes are responsible for the degradation of extracellular matrix (ECM) proteins, including collagen, laminin, and fibronectin, which contribute to ECM remodeling in various pathological processes [38]. Numerous studies have demonstrated that MMPs are highly expressed in solid tumors and are closely associated with cancer development and invasion [38,39]. Specifically, MMP9 is known to play a crucial role in the degradation of type IV collagen in the basal membrane surrounding the tumor, thereby promoting tumor cell infiltration and metastasis [21]. CD44 and MMP9 are frequently utilized as immunohistochemical markers that are concurrently evaluated in renal cell carcinoma (RCC) and have been identified as potential molecular prognostic markers for RCC [17]. In our previous study, it was observed that MMP9 was the sole MMP isoform among the 25 up-regulated genes that displayed heightened expression in ccRCC-TT. This observation suggests that MMP9 plays a crucial role in the metastasis of ccRCC. This study revealed that the expression of MMP9 was partially reliant on the CD44-HAS1 axis in clear cell RCC (ccRCC). The expression patterns of CD44, HAS1 and MMP9 in ccRCC-TT exhibited a similar expression tendency, indicating that the CD44/HAS1/MMP9 regulatory system may have a significant impact on the aggressiveness of ccRCC.

In summary, our findings demonstrate the crucial involvement of CD44-induced HAS1 expression in the regulation of MMP9 and the promotion of aggressiveness in ccRCC cells. This study offers novel insights into the impact of CD44/HAS1/MMP9 on ccRCC tumorigenesis and invasion, with potential implications for early metastasis prediction and the development of more effective treatment strategies.

## Data Availability

The data that support the findings of this study are available on request from the corresponding author.

## Supplementary Materials

The following supporting information can be downloaded at: https://www.mdpi.com/article/10.3390/biomedicines11072077/s1, Figure S1: CD44 overexpression promoted cell proliferation of A498; Figure S2: CD44 knockdown inhibited cell migration and invasion of 786-O; Figure S3: HAS1 was down-regulated in CD44 knockdown cells; Figure S4: HAS1 and MMP9 exhibited a correlation with the overall survival of ccRCC patients.
