# Supplementary material for: CD44 Is Associated with Poor Prognosis of ccRCC and Facilitates ccRCC Cell Migration and Invasion through HAS1/MMP9
Source: Biomedicines. 2023 Jul 24;11(7):2077. doi: 10.3390/biomedicines11072077 (PMC10377257; doi:10.3390/biomedicines11072077)
Supplement: Supplementary file 1 [file biomedicines-11-02077-s001.zip › biomedicines-2461215-supplementary.pdf]

Supplementary Materials

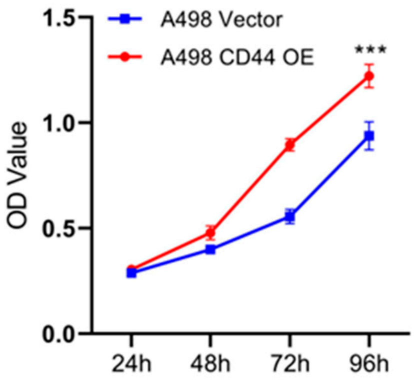

Figure S1. CD44 overexpression promoted cell proliferation of A498. *** $p < 0.001$. CCK-8 assay was performed to detect cell proliferation of A498 CD44 OE cells.

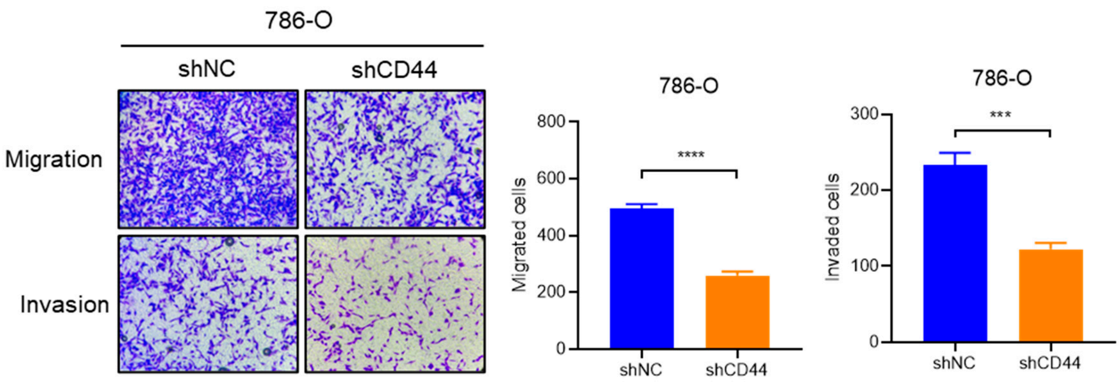

Figure S2. CD44 knockdown inhibited cell migration and invasion of 786-O. *** $p < 0.001$; **** $p < 0.0001$. Cell migration and invasion were analyzed using transwell migration and matrigel invasion assay.

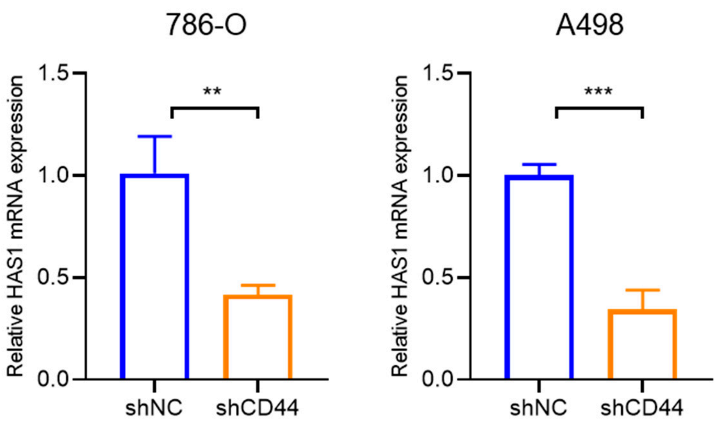

Figure S3. HAS1 was down-regulated in CD44 knockdown cells. ** $p < 0.01$; *** $p < 0.001$. qRT-PCR analysis showed HAS1 mRNA expression was decreased in 786-O and A498 CD44 overexpression cells.

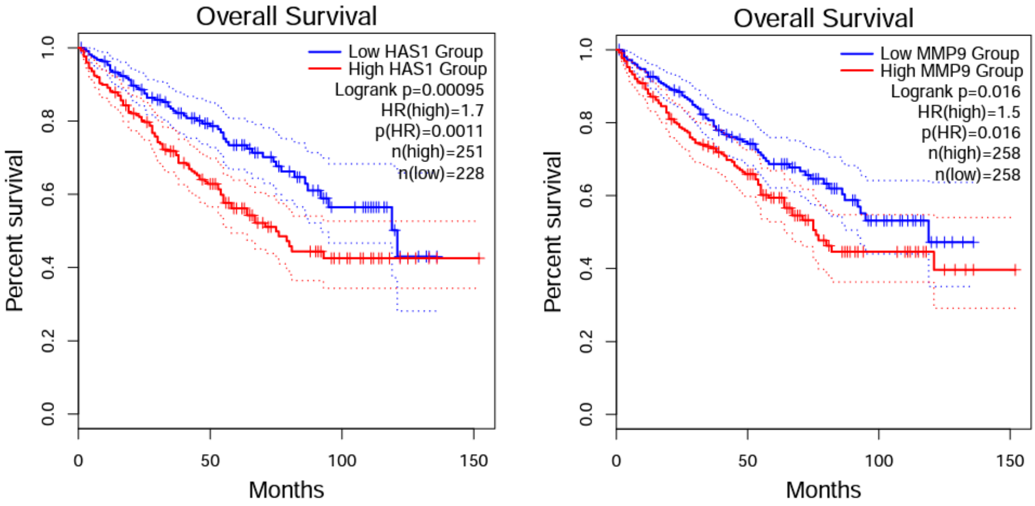

Figure S4. HAS1 and MMP9 exhibited a correlation with the overall survival of ccRCC patients. The Kaplan-Meier survival curves were utilized to assess the association between HAS1, MMP9 and the overall survival of patients with ccRCC in the TCGA-KIRC dataset.
